# The Spatial Similarity Task: A cross-species approach to spatial memory

**DOI:** 10.3758/s13428-026-03127-5

**Published:** 2026-08-18

**Authors:** Mia Borzello, William Supian, Andrea A. Chiba

**Affiliations:** Department of Cognitive Science, University of California, San Diego, 9500 Gilman Drive, Mail Code 0515, La Jolla, CA 92093-0515 USA

**Keywords:** Mnemonic discrimination, Spatial memory, Virtual reality, Cross-species

## Abstract

Memory serves as the cornerstone of cognitive function and specifically enables the critical ability to disambiguate and encode similar experiences as distinct memories. Mnemonic discrimination of near spaces and places is a process crucially underpinned by a computational feature of the hippocampus, pattern separation. Whereas this refined aspect of spatial memory has been addressed in abundance in the rodent literature, fewer spatial tasks are designed to specifically query memory for spatial similarities in human subjects. To address this, we introduce an open-source virtual maze suite developed on the Unity platform, freely available for researchers to use, modify, and extend. The suite is designed to bridge the behavioral translation gap by assessing mnemonic discrimination in humans through a spatial delayed match-to-sample task, carefully modeled after rodent spatial memory tasks. Rather than relying on fully immersive systems, the suite leverages desktop-based virtual navigation to combine parametric control of experimental conditions with the ability to collect each participant’s exploratory data. The pilot experiments are presented to validate the final suite design, alongside providing representative results from a subset of participants. Mnemonic discrimination was measured by parametric manipulation of spatial distances, resulting in a spatial distance memory function in which participants made more errors in remembering the correct location amongst similar or adjacent locations relative to distant locations. Given the prevalence of spatial memory deficits in age-related cognitive decline and neurological and psychiatric conditions, and the widespread use of spatial tasks in rodent models of these disorders, the Spatial Similarity Task (SST) suite offers a translationally grounded tool with direct potential for customization towards clinical application.

**Supplementary Information:**

The online version contains supplementary material available at https://doi.org/10.3758/s13428-026-03127-5.

## Introduction

Understanding the mechanisms of memory and cognition across species is a critical challenge in neuroscience. Memory organization is intricately dependent on both temporal and spatial dimensions, necessitating the disambiguation of memories with high degrees of overlap on one dimension or another. This disambiguation of similar experiences (i.e., highlighting the distinctiveness of each event or orthogonalizing them along some separating dimension) is the process by which overlapping memories are proposed to be distinguished from one another (Stevenson et al., 2020; Quian Quiroga, 2023). This process is most often referred to in neuroscience as mnemonic discrimination. The distinction between mnemonic discrimination and pattern separation as a computational and cellular process is critical. While mnemonic discrimination manifests as the ability to disambiguate similar experiences in observable tasks, it does not directly relate to the computational, cellular, or anatomical definitions of pattern separation as originally described by Marr (1971) and should not be interchangeably referred to under the umbrella term “pattern separation” (Stark et al., 2013). This nuanced understanding underscores the complexity of the process, highlighting the need to differentiate between its behavioral manifestations and its underlying computational and cellular mechanisms within the dentate gyrus of the hippocampus (Leutgeb et al., 2007).

While several tasks test aspects of spatial mnemonic discrimination ability (Liu et al, 2016; Leal & Yassa, 2018), the capacity to fully investigate this phenomenon is contingent upon the development of tasks that can reliably measure it in an analogous way across different species. Early efforts in this direction were pioneered by Kesner et al. (as cited in Gilbert et al, 2001) through the introduction of a task designed for rats, which was later adapted for human participants (Holden et al., 2012). Despite these advancements, the translation of findings from one species to another remains challenging. This is due, in part, to the inherent differences in the memory domain tested and the techniques employed, which can impede the scalability and translation of results across species (Schoenfeld et al., 2017) and may also reflect the wide individual differences in human navigational ability (Wolbers & Hegarty, 2010). Furthermore, ensuring behavioral relevance between species has emerged as a critical factor in translating the nuanced neuroscientific findings gathered from rodent work to bear relevance on human biomedical research.

These challenges motivated the development of the Spatial Similarity Task (SST), a spatial delayed match-to-sample task, designed to be comparable to tasks commonly used to investigate memory in rodents (Gilbert et al., 2001; Hunsaker & Kesner, 2013). The SST is designed to assess spatial memory and is hypothesized to engage hippocampal pattern separation mechanisms. While we have not directly demonstrated hippocampal dependence, the task is structured to allow for direct engagement of hippocampal circuitry (Doeller et al., 2010; Iglói et al., 2010; Kyle et al., 2015), and future work pairing the SST with neural recordings would help clarify the specific circuit contributions to performance.

The primary motivation behind the design of our task was to enhance the capacity to translate experimental learning and memory findings relevant to the functional integrity of the hippocampus, specifically the dentate gyrus, from rodents to human participant populations. These tasks generally involve varying degrees of spatial discrimination to assess mnemonic discrimination (Gilbert et al., 2001; Hunsaker & Kesner, 2013). See Table 1 for a comparison of the SST with prior human and rodent spatial memory tasks, highlighting key similarities in design and cognitive demands.

**Table 1:**
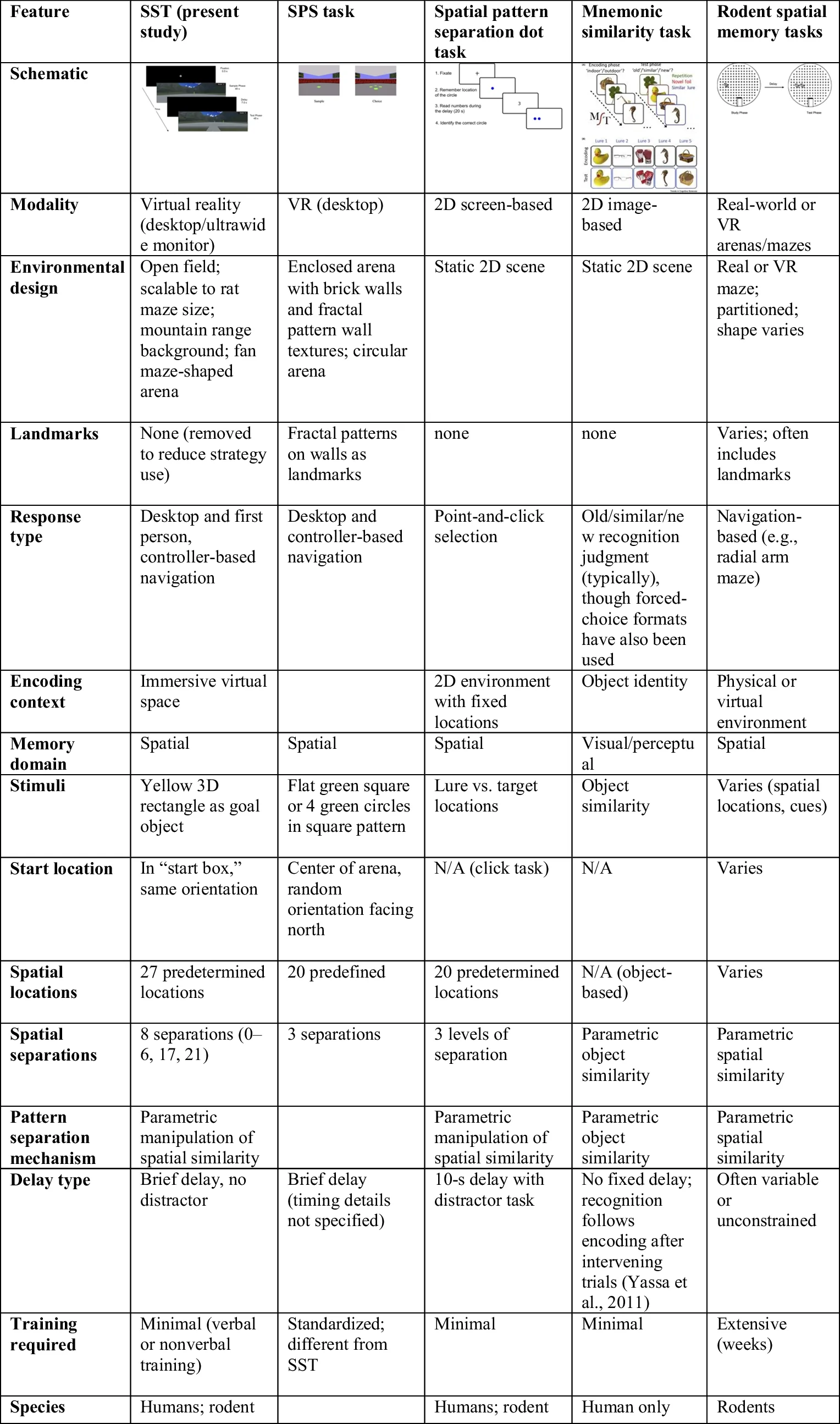

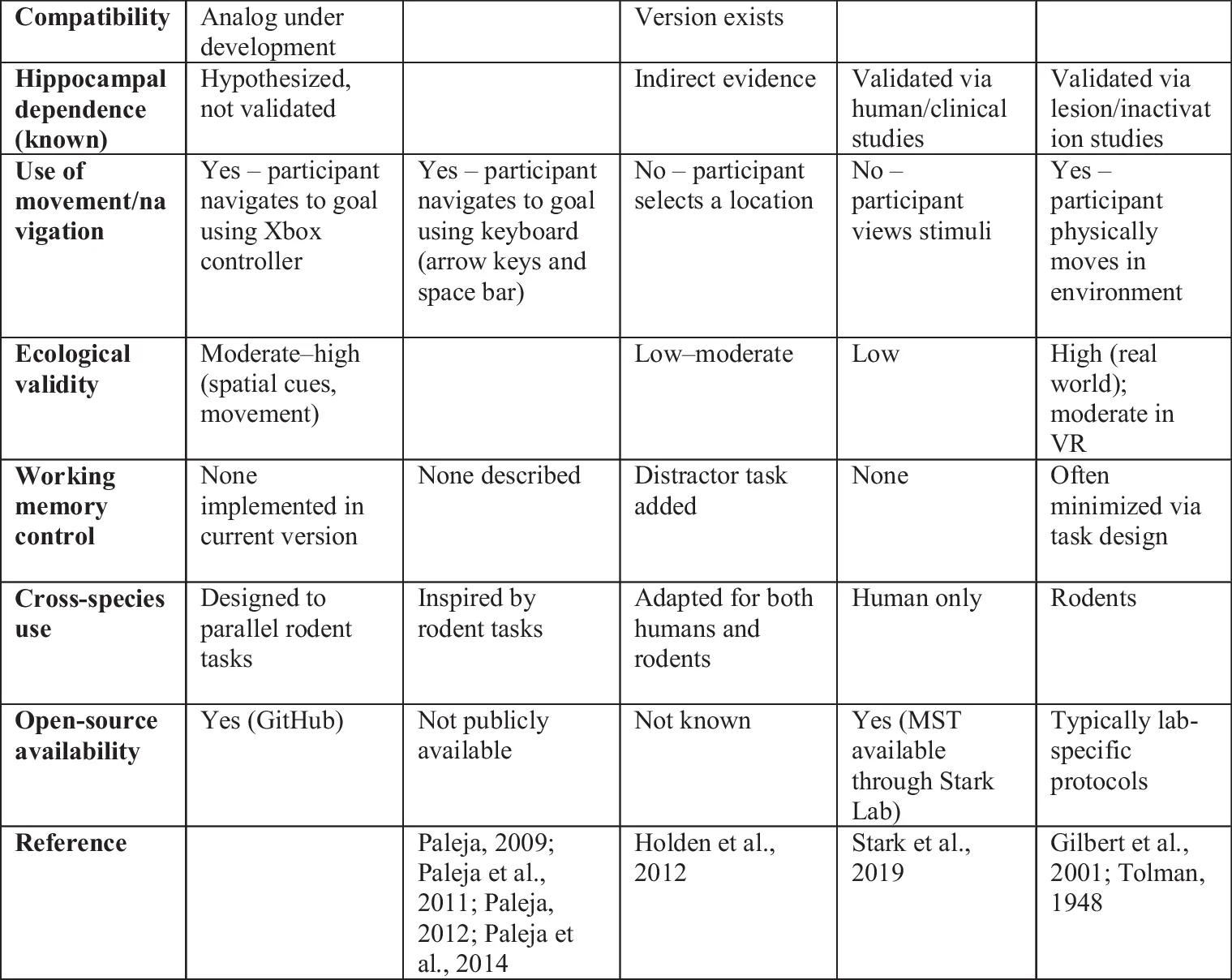
Comparison of the Spatial Similarity Task (SST) with prior human and rodent spatial memory paradigms. This schematic highlight key task affordances by comparing the SST to previous spatial memory tasks. The SST builds upon and differs from prior efforts in key areas including the environment design, spatial parameters, and navigational demands. Paleja et al.’s (2011) spatial pattern separation (SPS) task used a VR maze with visual landmarks and fewer spatial separations, whereas the SST removed such cues and introduced a start box, increased spatial locations, and integrated broader separation metrics to improve behavioral sensitivity and expand task affordances

While having humans navigate real spaces would serve as the closest homologue to rodent tasks, practical limitations typically prevent this approach. The SST therefore utilizes desktop-based virtual reality (VR) environments presented on standard and ultrawide monitors. In this manuscript, we use the term “virtual reality” to refer specifically to desktop-based navigation tasks presented on a monitor, distinguishing them from fully immersive systems that rely on head-mounted displays. While immersive systems may elicit stronger spatial presence, they also present challenges such as motion sickness and reduced accessibility (Diersch & Wolbers, 2019; Wiener et al., 2020). Desktop-based virtual environments provide a middle ground: they allow researchers to create highly controlled environments while still capturing richer navigational and spatial processes than traditional 2D computer-based tasks (Chrastil & Warren, 2012; Diersch & Wolbers, 2019; Thornberry et al., 2021; Kothgassner & Felnhofer, 2020; Pooja et al., 2024). Ecological validity in this context is relative rather than absolute. While not equivalent to real-world navigation, desktop navigation does engage spatial representations and strategies that are more naturalistic than static or non-navigational tasks (see also Kaufhold et al., 2026, for a broader discussion of the trade-offs between laboratory control and ecological validity in cognitive neuroscience).

Several research groups have developed sophisticated VR-based navigation and memory paradigms that have substantially advanced our understanding of hippocampal function, including ecologically rich virtual navigation tasks that have yielded important insights into allocentric and route-based spatial coding (Chrastil & Warren, 2012). However, many of these paradigms require specialized equipment, extended training, or technical infrastructure that may be impractical in clinical settings such as memory clinics or neuropsychological assessment suites (Stark et al., 2019; Alsbury-Nealy et al., 2022; Rekers & Finke, 2024; Vasser et al., 2017; Schuetz et al., 2023; Jangraw et al., 2014; Commins et al., 2020). Our suite was designed with clinical translatability as an explicit goal: it runs on standard desktop hardware, requires minimal experimenter training, and can be administered in under an hour. While we have not yet conducted a randomized controlled trial to validate the SST as a clinical diagnostic instrument, the flexible game architecture allows researchers and clinicians to independently manipulate the parameters most relevant to the cognitive constructs of interest, including delay duration (which modulates the temporal demands on hippocampal memory maintenance) and spatial distance between arm locations (which directly indexes the degree of mnemonic interference, and is thought to tap dentate gyrus pattern separation at close separations). This parametric flexibility is critical if spatial memory assessments are to be consistently implemented across laboratories, translated meaningfully across species, and ultimately adapted for use in clinical populations. The inclusion of the Spatial Pattern Association (SPA) task alongside the SST further demonstrates how the underlying framework can accommodate different task structures that probe related but distinct memory processes, and we anticipate that researchers may wish to develop additional variants, such as tasks probing temporal sequence memory or object–location binding, using this codebase as a foundation.

The parallel design of the SST for both rats and humans is particularly advantageous. It enables more direct comparisons across species, which is essential for probing the underlying mechanisms of spatial memory. By maintaining structural and procedural consistency between the tasks, the approach minimizes the variability that typically complicates cross-species research. This alignment is crucial for validating theories of cognitive function that span multiple biological models, thereby enhancing the robustness and applicability of the findings. To ensure methodological consistency across species, half of the human participants received explicit instructions about the task while the other half engaged in self-directed learning, mirroring the incidental learning process characteristic of rodent training, and addressing a critical methodological consideration in cross-species comparisons. This paper presents the design, implementation, and rationale behind our Unity-based desktop VR task aimed at assessing mnemonic discrimination and allowing comparisons across species. The subsequent sections delineate the design rationale and characteristics of the toolbox.

While the SST was designed to parallel aspects of rodent spatial memory tasks, we recognize that there are important differences between species-level implementations. Rats physically ambulate through the maze space, whereas humans interact with the task via controller-based navigation on a desktop display. Similarly, certain task design modifications were necessary to avoid ceiling performance in humans (e.g., removing maze arms, scaling the number of locations). These modifications reflect the broader reality of cross-species research; translation is rarely literal but instead involves constructing parallel task structures that preserve core cognitive demands while accommodating species-specific constraints, not unlike how a joke rarely survives word-for-word translation but can land perfectly in a different language if the underlying logic is preserved. Our aim is therefore not to claim a perfect equivalence between rodent and human implementations, but to create a framework that enables meaningful comparison across species by aligning methodological principles and parametric manipulations.

### Task suite overview and procedures

The Spatial Similarity Task is openly available and can be downloaded for free from the GitHub repository at the following URL link: https://github.com/Spatial-Similarity-Task. The suite is released under a Creative Commons BY-SA 4.0 license, enabling researchers to use, adapt, and build upon the framework for their own experimental needs. Example experiments, datasets, and accompanying MATLAB analysis scripts can be found on GitHub. We have recently used the task suite to investigate mnemonic discrimination across different memory domains in a sample of over 200 participants (manuscript under review). For broader theoretical background, see Hunsaker and Kesner, 2013.

For trial-level analyses of binary accuracy data, researchers may consider generalized linear mixed-effects models with binomial error structures (e.g., glmer in R), depending on their analytic goals.

**Fig. 1:**
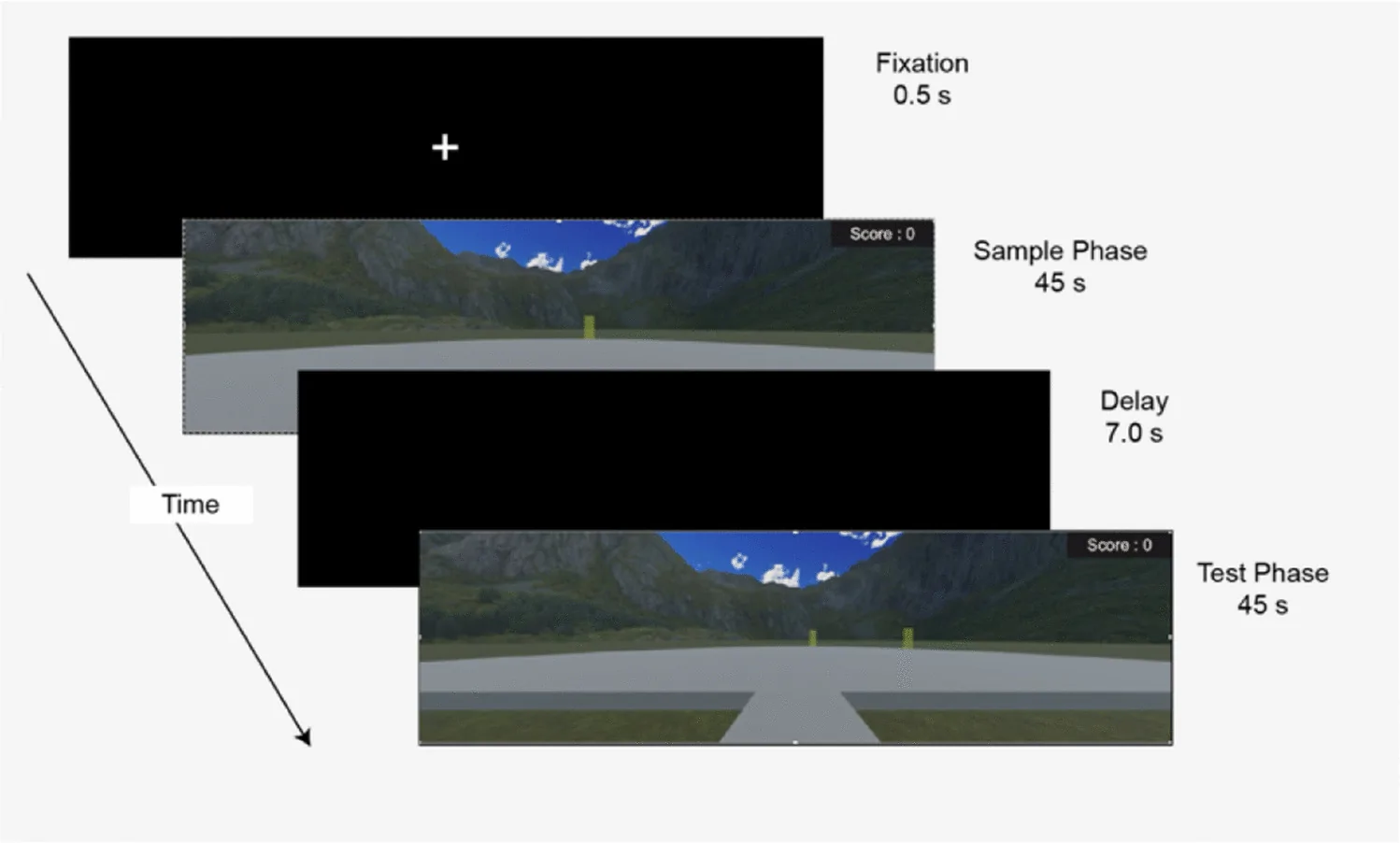
Spatial Similarity Task. This is a virtual maze environment. Each trial consists of a sample phase, a brief delay period, and a test phase

**Fig. 2:**
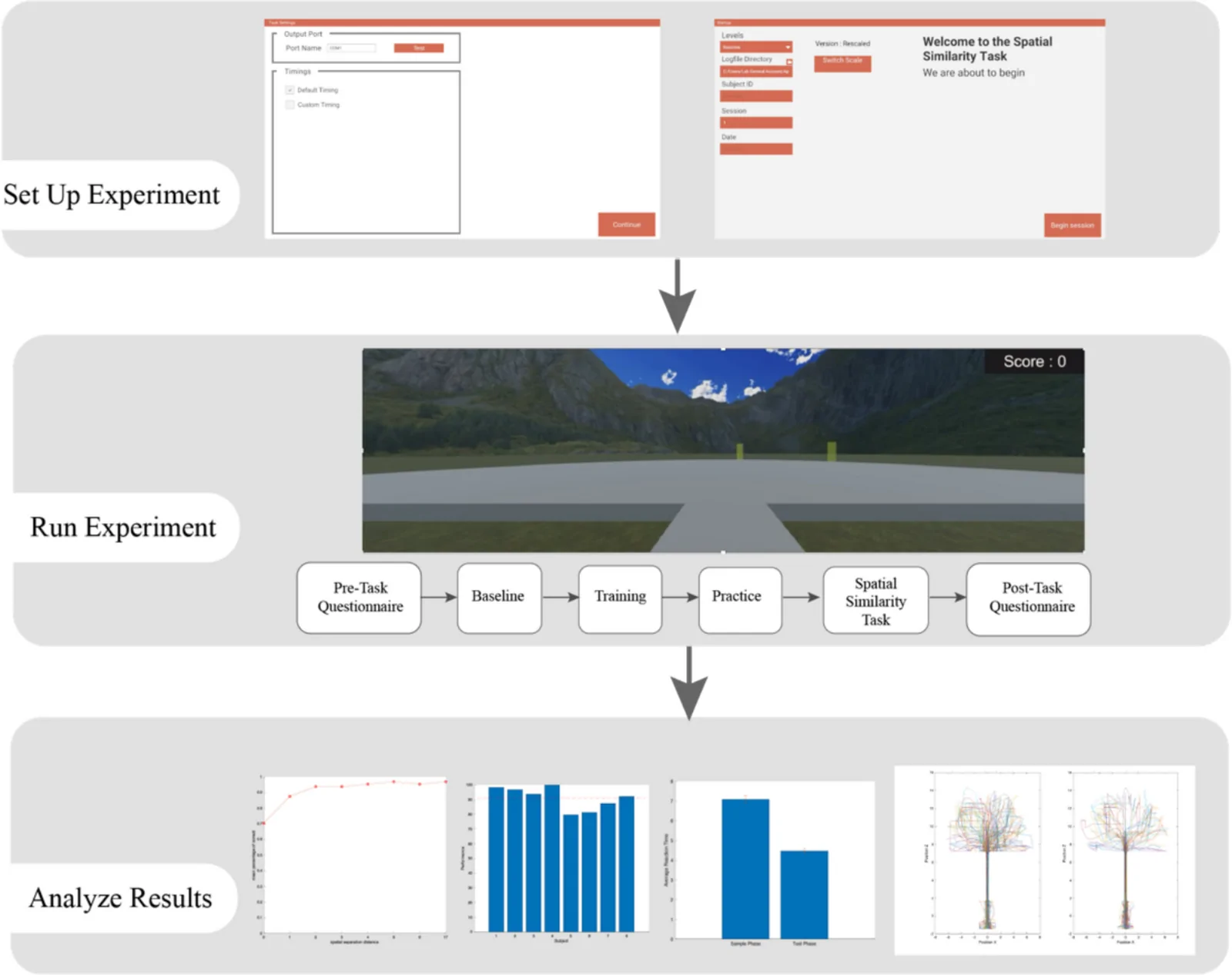
Overview of procedures from setup to analyzing the data

The present section provides an overview of task-specific experimental methodologies and manipulations, delineated within dedicated sections corresponding to the decisions the researcher will make in setting up the experiment and the protocol to run an experiment, outlined in Figs. 1 and 2 (see Supplementary Information for full protocol and Supplemental Fig. 1 for our lab setup). Methodological considerations, experimental recommendations, and parametric manipulations are discussed in turn.

### Spatial Similarity Task (SST)

The Spatial Similarity Task (SST; Fig. 1) is a delayed match-to-sample navigation task, adapted from the framework introduced by Gilbert et al. (2001) to examine mnemonic discrimination, and further informed by virtual maze tasks developed by Bohbot (Konishi & Bohbot, 2013) and Paleja (Paleja et al., 2011) to study aspects of spatial memory. In this task, participants learn a specific spatial location during the sample phase and are later required to identify that location when presented alongside a spatially adjacent foil during the test phase. The environment is rendered in a naturalistic virtual maze that enables the collection of fine-grained behavioral data, including path trajectories, movement speed, and viewpoint orientation.

Across 64 trials, participants navigated to targets randomly drawn from a pool of 27 predefined spatial locations arranged along the perimeter of the maze. Eight spatial separations were used (eight trials per separation), spanning a range of distances to assess different levels of spatial interference: separations of 0, 1, and 2 were categorized as “close”; 3, 4, 5, and 6 as “medium”; and 17 and 21 as “far.” These separation values are based on relative distances in the virtual space and were selected following extensive piloting to ensure a meaningful spread in task difficulty.

**Fig. 3:**
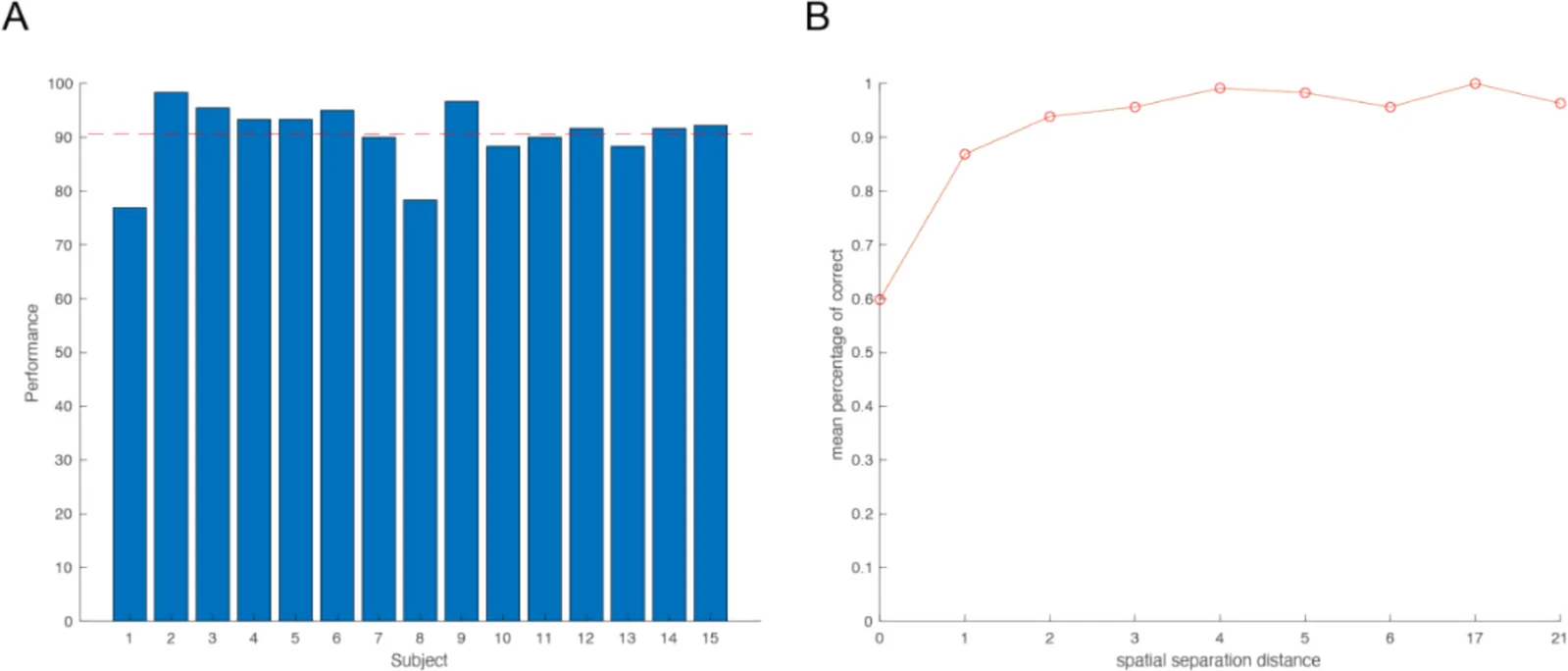
Group's data. **A** Performance of 15 participants shown with average performance as red dotted line. **B** Average accuracy as a function of spatial separation

**Fig. 4:**
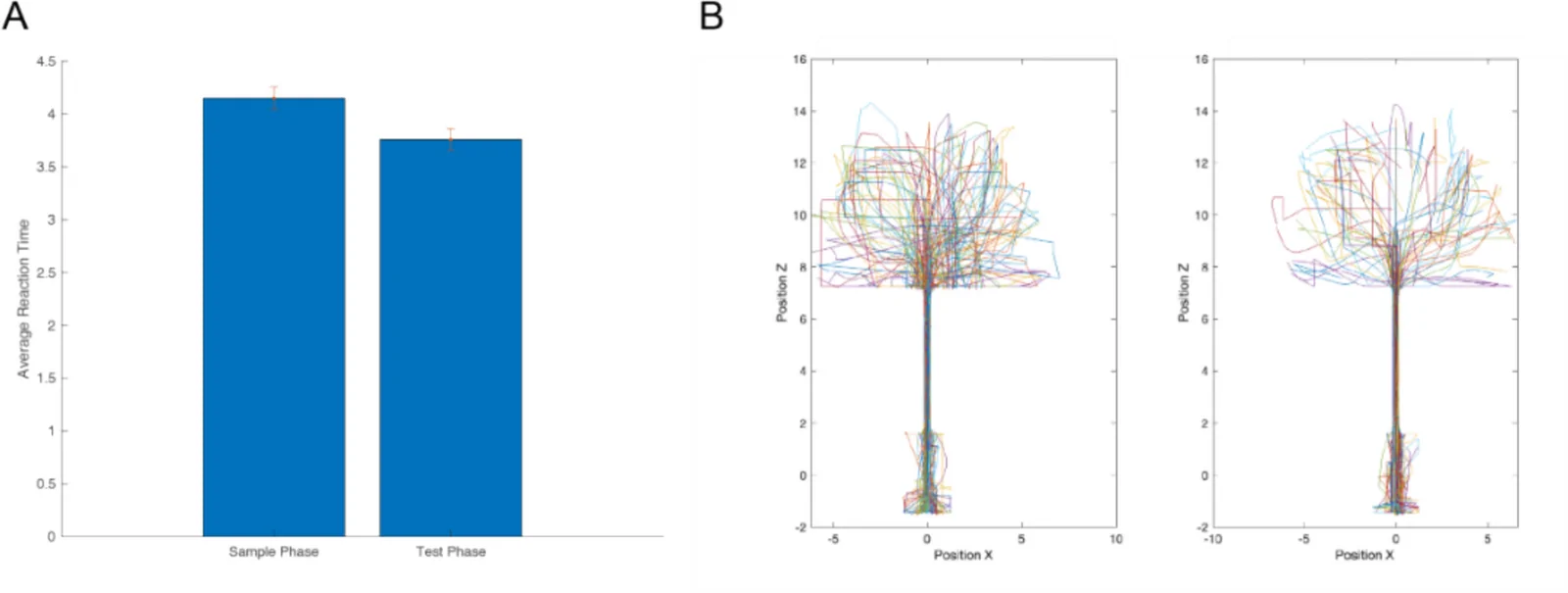
Single participant's data. **A** Average reaction time plot across both phases of a trial for one participant. **B** Plot of path trajectories for sample and test phases for a single participant

**Fig. 5:**
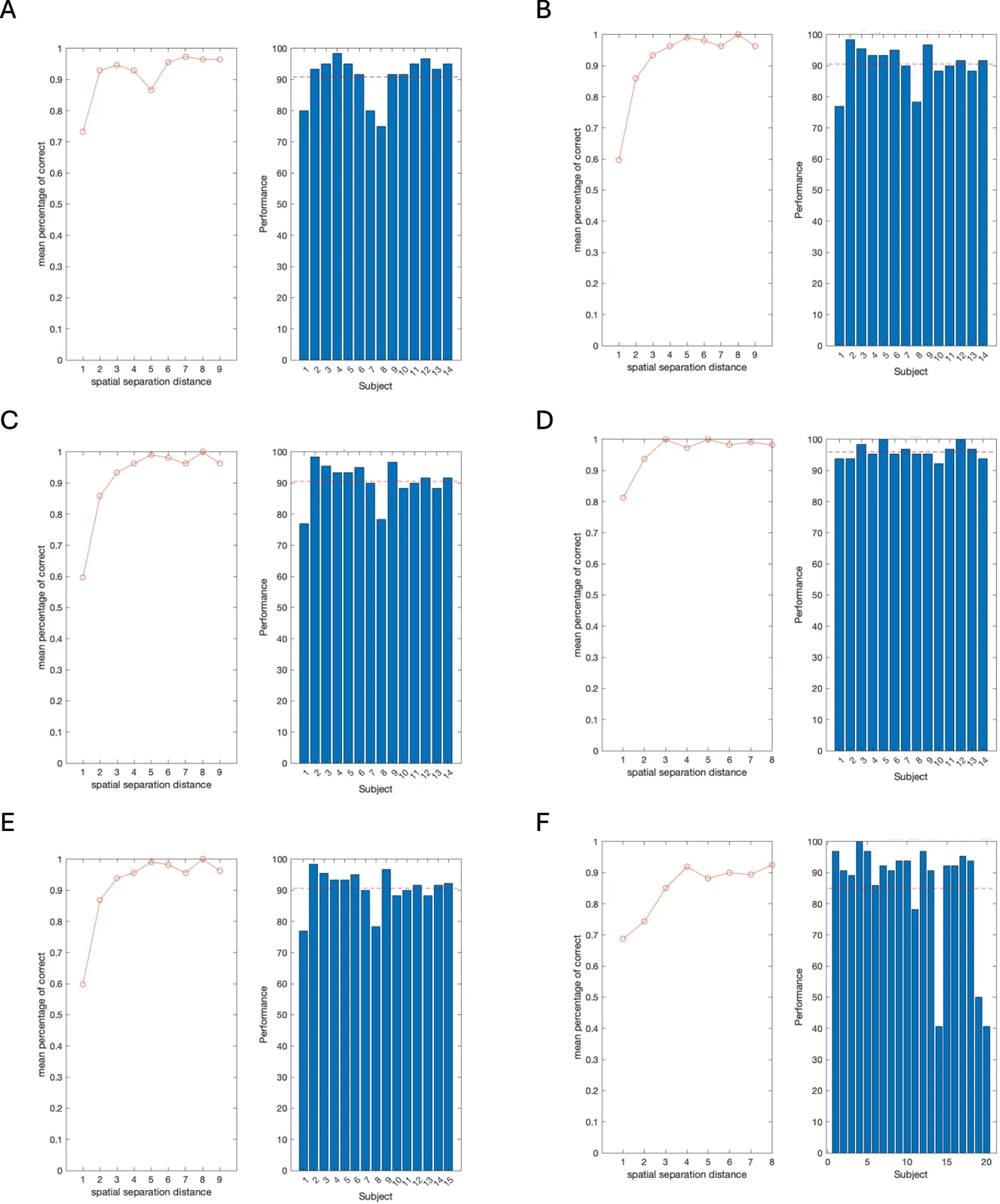
Comparison of different task design manipulations on accuracy as a function of spatial separation (left) and overall performance (right): Maze size (*n* = 14)- **A** original size maze, **B** rescaled rat-sized maze Monitor (*n* = 14)- **C** curved monitor, **D** flat monitor. Training type- **E** verbal training (*n* = 15), **F** nonverbal training (*n* = 20)

**Fig. 6:**
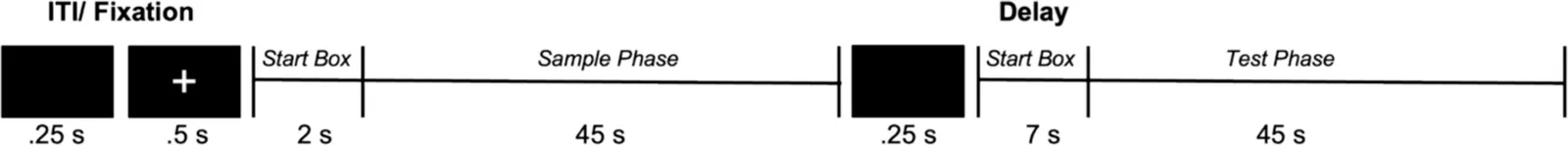
Trial structure of the Spatial Similarity Task (SST). Each trial begins with an inter-trial interval (ITI), during which a fixation cross is presented to orient the participant. After 2 s in the start box, the door opens and the sample phase begins, during which the participant navigates to a target object within the virtual environment. Following the sample phase, a 7-s delay occurs in the start box. The test phase then begins, during which the target and a foil object appear, and the participant must identify the original target location. Both the sample and test phases are self-paced, with a maximum duration of 45 s

Each phase of a trial (sample and test) lasted up to 45 s, separated by a fixed intermediate delay of 7 s to probe short-term spatial memory. Trials in which participants failed to respond within the 45-s window were scored as incorrect. Participants always began trials from the same starting location, mirroring the design of the rodent reference task to preserve cross-species comparability. The trial structure, including the sample and test phases and the intermediate delay, is illustrated in Figs. 3, 4, 5 and 6.

While we used a single, intermediate delay of 7 s, future work, particularly in patient populations, would benefit from imposing multiple delay durations (e.g., 1, 7, 30 s) to more thoroughly probe the temporal dynamics of memory retention. Prior work has shown that the hippocampus may support short-term memory across even brief delays, particularly when memory load is high or relational information must be retained (Jeneson & Squire, 2012; Jeneson et al., 2011). The flexibility of our task parameters supports delays of varying lengths, a design choice that becomes especially consequential when comparing across age groups or clinical populations, where the threshold between working memory and long-term memory engagement may differ substantially.

### Spatial Pattern Association (SPA) task

The SPA task (Supplemental Fig. 6) used in this study builds on the virtual navigation paradigm introduced by Paleja (2009), which was developed to assess binding of object–location (place) associations (Kesner & Hopkins, 2006) using a delayed recall format. A detailed comparison of the present SPA task and the version reported by Paleja et al. (2011) is provided in Table 2, highlighting methodological and structural similarities and differences.

**Table 2:**
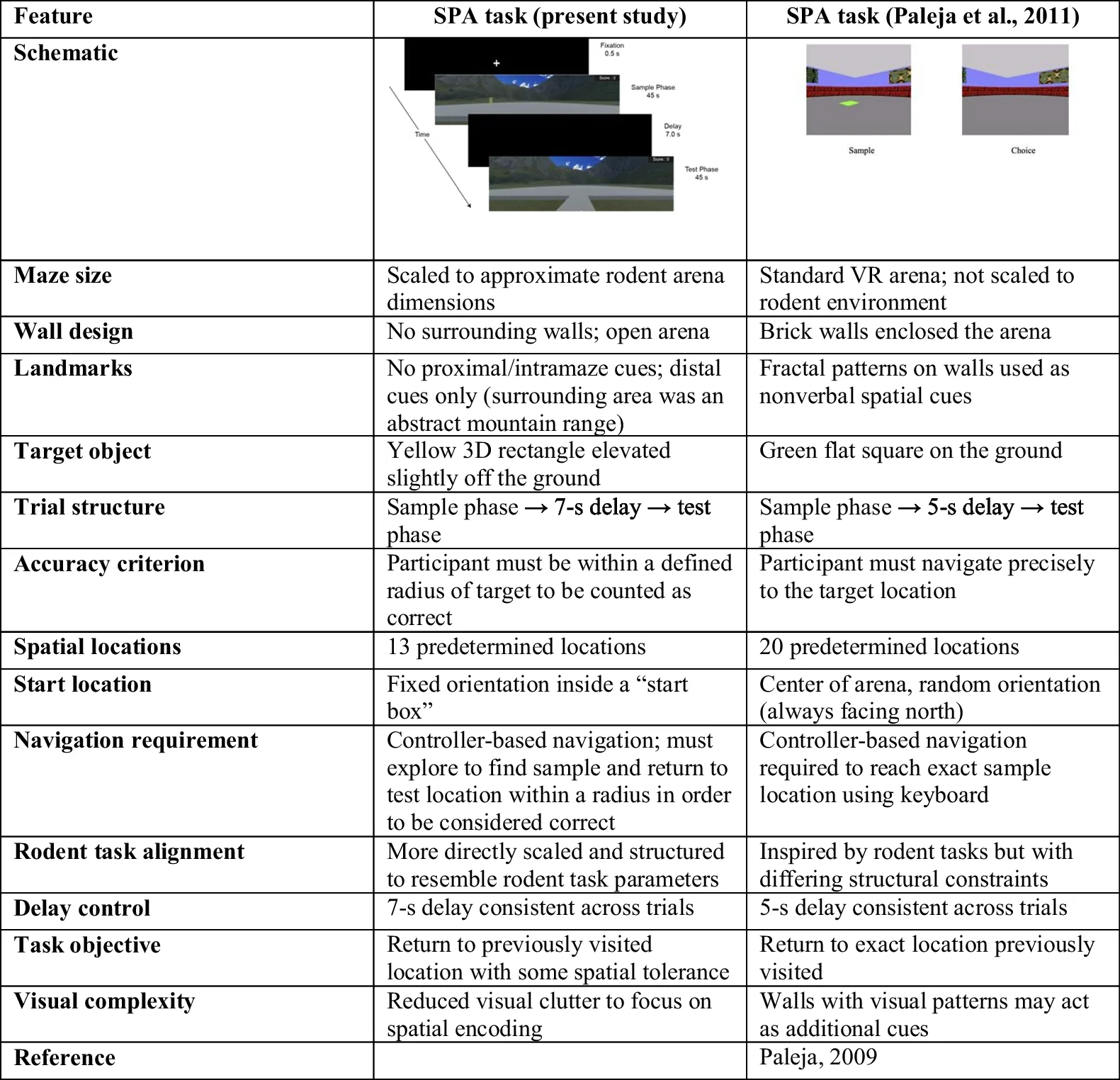
Comparison of the SPA task developed in the present study with the SPA task reported by Paleja et al. (2011). While both tasks aim to assess spatial associative memory using virtual navigation, the present version builds on the Paleja task design and was scaled to align more closely with rodent task parameters. The updated task removed external visual cues to isolate spatial memory demands and included additional modifications such as extended delay intervals, altered spatial tolerance criteria, and changes in environmental setup

While the SPA shares some structural elements with the SST, it introduces a key difference: during the test phase: participants must retrieve the remembered target location without local, visual objects present, making it a recall-based task rather than one dependent on recognition. Although the environment did not contain proximal or intramaze objects, distal cues (i.e., surrounding mountains) were available, and participants could in principle use beaconing or piloting strategies based on these cues. This distinction between tasks anchored by proximal intramaze objects and those relying solely on distal extramaze cues parallels design choices made in foundational rodent spatial memory paradigms such as the Morris water maze (Morris, 1984), and is worth bearing in mind when interpreting performance differences between the SST and SPA. The environment consists of 13 prespecified target positions arranged along the perimeter of a fan-shaped maze. Participants completed 64 trials, with each phase of the task lasting up to 45 s, separated by a 7-s delay to probe short-term spatial memory. We also calculated a distance error measure (i.e., the Euclidean distance between the correct location and the recalled location), consistent with prior approaches (e.g., Doeller et al., 2010; Moon et al., 2022; Beanato et al., 2024).

As with the SST, failing to respond within the allotted time resulted in an incorrect score for that trial. This design enables the measurement of participants’ ability to recall spatial locations in the absence of supporting object cues.

### Parametric manipulations: Setting up the experiment and testing participants

#### Methodological considerations

Tasks conducted with rats and those with humans exhibit fundamental differences due to intrinsic dissimilarities in instruction and interaction with experimental setups. These differences are not incidental; they reflect species-specific constraints that must be acknowledged rather than minimized if cross-species comparisons are to be meaningful. As noted in the Introduction, the SST is modeled on rodent paradigms but necessarily diverges in key respects. Design adjustments reflect the need to calibrate task difficulty for human participants. Acknowledging such divergences is essential. Translation across species depends not on identical surface features but on the preservation of shared cognitive demands (e.g., interference resolution under varying spatial separations). By making these parallels and limits explicit, we situate the SST as a cross-species parallel task that maintains core methodological principles while accommodating species-specific constraints.

1. *Training methodology*: The primary divergence lies in the mode of training. In rodent tasks, learning typically occurs through repeated exposure to stimuli and reinforcement over time, without the use of language. This nonverbal training unfolds gradually, often over months, and requires extensive habituation and shaping of behavior through repeated interaction with the task environment (e.g., via rewards or exploratory learning). In contrast, human participants are typically given verbal instructions, enabling a more immediate grasp of the task objectives through explicit scaffoldings. This distinction should not be taken to imply that rodents operate solely on “instinctual responses,” a framing that underestimates their capacity for flexible learning and spatial inference. On the contrary, foundational work such as Tolman’s (1948) theory of cognitive maps (Duvelle & Grieves, 2026) has long challenged such assumptions, demonstrating that rats can form internal representations of space and adapt their behavior accordingly. Indeed, when stimulus–response learning does occur in rodents, it is thought to depend on striatal circuits, particularly the basal ganglia, rather than the hippocampus (Packard & McGaugh, 1996), underscoring that the distinction we draw here is mechanistic as much as procedural. The relevant difference here is not one of capability, but of communicative context and procedural constraints: humans can be briefed and queried in minutes, whereas rodents must come to understand task contingencies incrementally. This contrast reflects less a hierarchy of cognitive sophistication than a divergence in how learning must be facilitated across species. The impracticality of training humans in a manner akin to animals underscores the necessity for distinct methodologies.
2. *Time constraints on trials*: Another critical difference is the temporal structure of the trials. Trials in rats are typically unconstrained in time, allowing for natural exploratory behavior and cognitive map formation. In contrast, humans are often given a fixed time limit for each trial, introducing time pressure that can affect strategic planning and decision-making, thereby altering task engagement.
3. *Physical configuration of the maze*: The structural setups for rat and human tasks vary significantly due to practical constraints and species-specific requirements. Rat tasks, including mazes, are often constrained by lab space limitations, resulting in proportionately smaller and more confined experimental setups with arm partitions that guide movement paths, essential for studying spatial memory and decision-making in a controlled environment. In contrast, human tasks are more visually oriented and can leverage virtual environments to create larger, more intricate, and richly detailed setups that support controlled navigation and spatial learning. The SST leverages this capacity: multiple spatial locations allow adjustment of the proximity between sample and target, facilitating measurement of both working memory and mnemonic discrimination, and the parametric distance function that results is precisely what enables meaningful comparison with rodent data. These distinctions are not just methodological but are also reflective of the differing cognitive demands and behavioral repertoires of the two species. Research on mnemonic discrimination must adhere to two crucial criteria: parametric modulation of interference between stimuli, and establishment of a behavioral response function that correlates with the level of interference. Additionally, it is essential for tasks assessing mnemonic discrimination to contain a distinct encoding phase, as this inherently involves encoding information in a way that reduces interference between memory representations or stimuli, thereby enhancing subsequent retrieval (Hunsaker & Kesner, 2013). Getting these design elements right matters; a task that lacks parametric structure or collapses the encoding and retrieval phases loses precisely the sensitivity needed to detect mnemonic discrimination effects.

#### Experimental recommendations and manipulations

To set up the experiment, the experimenter must decide on the total number of trials and the arm separations to use in the task. The SST task has 27 possible target locations on the maze. This means there are 25 separations that can be tested with 702 possible arm combinations. As a frame of reference, the authors collected 64 trials and used arm separations 0–6, 17, 21 (see Supplemental Fig. 2).

There are a handful of other manipulations the experimenter can take advantage of such as choosing the training type (verbal versus nonverbal training), maze design (maze with or without arms), maze size (rat-sized maze and larger environment), and the monitor to use (flat or more immersive monitor). Parameters such as trial timing (see Supplemental Fig. 5) and the specific set of separations to be tested can also be configured. These adjustments can be made directly through the graphical user interface shown in Supplemental Fig. 5. A summary of all configurable parameters and their available options is provided in Supplemental Table 1. The codebase is organized in a modular fashion so that users with programming experience can replace or extend components (e.g., modifying the maze environment, adding new object textures, or altering scoring criteria) without rewriting the core framework. This modularity reflects our broader goal of providing an open platform that can evolve with the needs of the research community. The inclusion of the SPA task within the same suite illustrates this capacity: it shares the core navigation and data-logging infrastructure with the SST but implements a distinct recall-based memory demand. Researchers interested in probing other memory domains, such as temporal sequence memory, object–context binding, or route learning, can build upon this foundation. We view the current release as a starting point rather than a finished product, and we encourage users to contribute modifications or new task variants back to the community. This level of flexibility allows the task suite to be adapted across a wide range of populations and experimental goals. For example, patient populations may require longer trial durations or reduced similarity between targets and foils, while studies in healthy young adults may benefit from tighter timing constraints or an emphasis on highly similar separations.

A central strength of the SST architecture is its capacity to manipulate the two parametric dimensions most relevant to hippocampal memory processing: delay duration and spatial distance (arm separation). These are not arbitrary design choices but reflect theoretically motivated links to distinct neural mechanisms. Delay duration modulates the temporal demands placed on memory maintenance; prior work suggests that even brief delays can recruit hippocampal circuitry when relational information must be held across an unfilled interval, with longer delays increasingly differentiating hippocampal from non-hippocampal contributions to performance (Jeneson & Squire, 2012; Jeneson et al., 2011). Spatial distance directly indexes the degree of pattern overlap between memory representations: closer arm separations create highly similar spatial inputs that require dentate gyrus-mediated pattern separation to be successfully disambiguated, while larger separations reduce interference and presumably engage pattern completion mechanisms in CA3 (Leutgeb et al., 2007; Hunsaker & Kesner, 2013). Importantly, very small differences in task design, such as whether the delay is filled or unfilled, or whether separation is defined in allocentric or egocentric space, can selectively engage different subregions of the hippocampal formation. The modular architecture of our suite allows researchers to independently vary these parameters, providing a flexible framework for dissecting the contributions of specific neural circuits to spatial memory across species and populations.

The significance of conducting preliminary experiments is paramount. Flaws in experimental design can result in spurious results, such as false-negative or false-positive outcomes. For common implementation issues and solutions, see Supplemental Table 2.

For each participant, we collect questionnaire data—pre-task, post-task, Navigation Strategy Questionnaire (NSQ), and Santa Barbara Sense of Direction Scale (SBSOD)—and data from the SST. The pre-task questionnaire collects demographic information (sex, age, education, handedness, first language) as well as medical history (brain injury, psychiatric and neurological disorders, medications) and day-of factors (fatigue, dizziness, recent food, alcohol, tobacco, or sleep disruption). The post-task questionnaire assesses previous video game and virtual reality experience and probes navigational strategy (e.g., “In relation to your final trial, please describe the strategy you used to locate the goal. What parts of the environment did you use, if any; how did you start searching, etc.”). The NSQ is a 14-item questionnaire that assesses an individual's affinity for cognitive map-based strategies when navigating (Brunec et al, 2018). The SBSOD measures spatial ability and is commonly associated with navigational performance in both real-world and virtual environments (Hegarty et al., 2002). Questionnaires have been included in the Supplementary Information.

### Measured parameters

The data recorded for each participant comprise two file types:

1. An overall session CSV file for the session and
2. Trial-by-trial data stored as one CSV file per trial (Fig. 3).

This is true for both the practice module and the SST task module. These files are saved in a folder labeled with the participant’s ID code (assigned at the start of the session) and the recording date (see Supplemental Fig. 3 for an overview of the folder structure).

The overall CSV file logs the following measures (Table 3): trial number, target arm, lure arms, the participant’s response, the sample phase reaction time, the duration of the sample phase duration, the test phase reaction time, and the duration of the test phase. Each trial-level CSV logs the following (Table 4): time in milliseconds, posX, posZ, the on-screen event, the phase of the trial, and controller input. Examples of both CSV files are shown in Supplemental Fig. 4. These variables are described in Tables 3 and 4.

**Table 3:** Methodological recommendations for adapting the spatial task suite across populations. Recommended modifications to the SST task for adapting the task suite to specific research questions or populations. These adjustments allow researchers to tune the difficulty, isolate cognitive processes, and enhance sensitivity to group differences

Research goal/use case (population or purpose)	Modification	Effect on task difficulty	Rationale/purpose
Reduce task load (e.g., for aging populations, young children, early-stage decline, or impaired populations)	Increase number of far separation trials	Decrease	Eases difficulty by maximizing distance between targets and foils
Detect subtle memory impairments (e.g., for high performers)	Increase number of close spatial separations (e.g., 0–2)	Increase	Boosts task sensitivity by increasing spatial interference and fine-grained discrimination demands
Probe memory retention over time	Extend delay between sample and test phases	Increase	Challenges short-term retention; reveals memory decay over time
Lower working memory demand	Shorten delay interval	Decrease	Reduces working memory demands and task load
Isolate hippocampal function from working memory	Add a concurrent task during delay	Increase	Minimizes influence of working memory rehearsal strategies and isolates hippocampal-dependent encoding and retrieval
Increase executive demands	Reduce response time window	Increase	Adds time pressure; emphasizes rapid decision-making and planning

**Table 4:** Raw data from session

Trial_num	Trial number
Target_arm, lure_arm	Arm combination for each trial- target and lure arms
Subject_response	Participant response for each trial (1 = correct, 0 = incorrect)
sample_phase_duration	Time from when start box door opens to when participant returns to start box in sample phase
sample_phase_choice_duration	Time from when start door opens to when participant makes choice in sample phase; sample phase reaction time
test_phase_duration	Time from when start door opens to when participant returns to start box in test phase
test_phase_choice_duration	Time from when start door opens to when participant makes choice in test phase; test phase reaction time

Beyond these raw logged variables, the following measures can be derived: session accuracy, arm separation used in each trial, accuracy as a function of arm separation, the participant’s trajectory in each phase of the trial, path length, and dwell time. Together, these outputs support both coarse performance summaries and fine-grained analyses of navigational behavior, the latter being one of the genuine advantages of a virtual environment over traditional pencil-and-paper or static screen assessments. A screen recording is also captured during the SST task. Custom-MATLAB scripts for analyzing the data and reproducing the plots in Figs. 4 and 5 are available on GitHub (https://github.com/Spatial-Similarity-Task).

### Validation experiments

An important aspect of the development of this task was the piloting stage. Piloting lasted several months and was performed in stages such that we could test performance on different conditions. The maze design itself underwent several iterations. In collaboration with a research laboratory at the University of California, San Diego (UCSD), the lead author collected data on a novel spatial associative learning task (adapted from Gilbert et al., 2001) that requires rats to learn an initial location as distinct from others in order to return to the location at a subsequent time to receive a reward (spatial delayed match to sample task; (Borzello et al., 2021). The original virtual maze design mimicked the rat maze; however, modifications were necessary to scale the task appropriately for human participants. In the end, the authors created a tightly linked virtual task. The final version of the SST task is scaled for size according to the relative size of the rat open arena.

#### Early piloting: Maze design, target locations, and landmarks

Initially, the virtual maze featured solid-colored walls between each target location, closely mimicking traditional rat maze designs. Participants performed at ceiling levels under these conditions, prompting us to adjust the design. We first made the walls transparent, allowing participants to see from one arm of the maze to another. Despite this change, performance remained at ceiling levels, leading us to remove the walls entirely, resulting in an open-field maze design.

The number of target locations or arms was another critical variable. We began with 14 locations but increased this to 27 after observing ceiling effects in participant performance. This modification aimed to enhance task difficulty and better capture variations in spatial learning and memory.

With the maze now comprising 27 locations, we explored the effect of arm separation on performance. A vast number of 702 combinations were systematically piloted, and detailed configurations are provided in the Supplementary Information.

Early iterations of the maze included salient landmarks such as buildings, a windmill, and a hot air balloon. These visual cues, however, led to participants verbalizing their strategies, thereby shifting the task away from pure spatial navigation. Consequently, landmarks were excluded from the final version to maintain the task's spatial nature.

#### Experiment 1: Effect of maze size on spatial learning

To investigate the influence of maze size, we created two versions: one matching the dimensions of a typical rat maze, scaled up using the rat-to-human ratio, and a larger version. Participants were tested on both mazes in a within-subjects design. A subset of this data is shown in Fig. 5A and B. A paired *t*-test revealed no statistically significant difference in overall performance between the two conditions (*p* = 0.817; 95% CI for mean difference, −2.493 to 3.106). These findings suggest that maze size did not significantly influence performance outcomes in this task, highlighting the robustness of the memory task performance across varying maze sizes. Notably, performance on the larger maze approached ceiling more rapidly across separation levels, likely because the rat-scaled maze produces smaller, and therefore less perceptually distinct, distances between locations. This experiment also highlights the advantage of virtual reality (VR) tasks, where scalability of space is not a limiting factor. Traditional rodent tasks are often constrained by the availability of lab space, resulting in proportionately smaller mazes; virtual tasks are not, allowing the environment to be tailored more directly to the research question.

#### Experiment 2: Effect of display type on spatial learning performance

The SST was tested on both standard flat screen monitors and ultrawide, curved monitors. Prior work has shown that the platform on which a spatial task is experienced can shape navigational strategy; participants adopt different approaches depending on whether they complete a task on a standard monitor or a VR headset (Clemenson et al., 2020). The present experiment examined whether display type similarly influenced performance on the SST. Participants were divided into two groups, one using a 49″ ultrawide, bent/curved monitor and the other a 19″ flat monitor. While initial observations suggested minimal performance differences, the ultrawide monitor induced motion sickness in some participants. Statistical analysis revealed a significant performance advantage for the ultrawide, bent monitor (*p* = 0.005; 95% CI, 1.795 to 9.115), suggesting that its broader visual field and increased sense of spatial presence confer a performance benefit. These findings suggest that the ultrawide, bent monitor enhances task performance, likely due to spatial advantages conferred by its broader visual field and increased sense of spatial presence. However, this immersivity came with the drawback that several participants reported motion sickness, with some unable to complete the task. The ultrawide, bent monitor is also considerably more expensive than a standard flat monitor, which is an important consideration for researchers when designing their studies. These trade-offs are worth bearing in mind: the ultrawide monitor may be preferable for studies prioritizing spatial sensitivity, while the flat monitor offers greater accessibility and participant comfort. Further analysis incorporating the complete dataset is recommended to confirm and extend these findings. Results are shown in Fig. 5C and D.

#### Experiment 3: Examination of instructional versus instrumental learning

This experiment compared spatial learning outcomes between participants receiving explicit verbal instructions (“instructional learning”) and those engaging in self-directed, nonverbal training (“instrumental learning”), an approach that parallels the learning paradigms used with rats, enhancing cross-species methodological alignment. Participants in the two conditions were distinct groups; no individual completed both training types, ensuring that comparisons were not confounded by within-subject learning effects. Our findings indicate that participants trained with nonverbal demonstrations performed comparably to those given explicit verbal instructions (Fig. 5E and F). This suggests that nonverbal training can serve as a viable and ecologically meaningful approach, aligning more closely with behavioral shaping and training in animals and helping to minimize potential confounds introduced by language or higher-order cognitive strategies. An independent-samples *t*-test revealed no statistically significant difference in performance between conditions (*p* = 0.264; 95% CI, −4.462 to 15.737). Rather than treating this null result as a non-finding, it is worth emphasizing what it affirmatively demonstrates: nonverbal training is a viable and ecologically meaningful approach that produces comparable performance to explicit verbal instruction. This alignment is consequential for cross-species work, where the absence of language-based instruction is a given rather than a choice. By showing that verbal scaffolding is not necessary for task acquisition, the SST preserves one of the core procedural parallels with rodent paradigms. Results are shown in Fig. 5E and F.

## Representative results

Figure 3A presents the group-level analysis from 15 participants, including both individual data points and average accuracy across spatial separations. Figure 4 shows data from a single participant. The raw data from these 15 participants has been made available on GitHub.

As seen in Fig. 3B, performance is notably lower at the closest separations (0), where target and foil objects are spatially adjacent—a high-interference condition. Lower performance on close separations indicates sensitivity to interference. This drop in performance at close separations suggests that the SST is successfully engaging interference resolution processes, a core behavioral hallmark of mnemonic discrimination. In trials with high spatial similarity, participants must disambiguate overlapping memory representations, an ability thought to depend on hippocampal computations that reduce interference between similar inputs. In contrast, accuracy for medium and far separations (1–21) is uniformly high, with performance exceeding 90% across most of these conditions. This pattern indicates that the task is more easily performed at moderate and far separations by healthy young adults, resulting in ceiling-level accuracy that reflects the expected robustness of spatial memory in this population. While this limits sensitivity to individual differences at these distances, it also provides a clear baseline from which task difficulty can be adapted for other populations or research goals.

The SST yields high accuracy across medium and far separations in healthy young adults, with performance approaching ceiling. This stability provides a useful baseline for establishing normative performance, but it also limits the task’s sensitivity to individual differences at these distances. Ceiling performance should therefore not be viewed as “optimal,” but rather as a constraint to be considered when applying the task across populations. Importantly, sensitivity to interference is preserved at the proximal or closest separations (0), where performance reliably drops, allowing the SST to detect fine-grained spatial memory demands. In contrast, the SPA task is generally more difficult, with no ceiling effects observed in the authors’ pilot studies, making it well suited for use in populations with higher baseline memory performance or in studies targeting fine-grained spatial recall. Together, these two tasks offer complementary sensitivity profiles—the SST anchoring the recognition end of the spectrum, the SPA the recall end, giving researchers flexibility in matching task demands to their population of interest. To facilitate the use of the SST task across a range of research contexts and participant populations, Table 5 outlines methodological modifications for adapting task difficulty. These adjustments can help fine-tune the task suite for use in studies of aging, clinical populations, or children, and support the flexibility of the task suite for investigating memory precision, interference resolution, and spatial learning in populations with different cognitive profiles. For example, the task can be made more challenging by increasing the delay between the sample and test phases, incorporating more trials with close spatial separations, or reducing the response time window. Conversely, it can be simplified for older or more impaired participants by reducing spatial similarity between targets and foils, shortening delays, or allowing extended trial durations.

**Table 5:** Trial-by-trial data from session

time	Time in seconds
posX	The participant's movement along the *x*-axis
posY	The participant's movement along the *y*-axis
event	Event in task (e.g., whether prompt is showing or if participant is performing the task)
phase	The phase of a trial (sample, test)
Input	Logs Xbox controller inputs and button presses

## Discussion

In this paper, we describe an open-source navigation tool designed to serve as a flexible, modifiable platform for researchers looking to probe aspects of spatial memory. It is worth underscoring a principle that motivates much of our methodological discussion: small, seemingly minor variations in task design can have outsized consequences for which neural systems are recruited. The presence or absence of a delay, the length of that delay, whether navigation is allocentric or egocentric, whether objects serve as landmarks or targets—each of these factors can shift the balance of contributions from the hippocampus, entorhinal cortex, striatum, or prefrontal cortex (Iaria et al., 2003; Packard & McGaugh, 1996). This sensitivity to parametric variation is precisely what makes cross-species translation challenging, and what makes a flexible, parameterizable task architecture scientifically valuable.

There remains a need for platforms that researchers can adapt to their specific experimental questions while maintaining methodological consistency across laboratories and species (Stark et al., 2019). We detail the software’s current architecture and a protocol for conducting a behavioral experiment using a virtual spatial memory task. The present protocol provides the necessary steps for researchers who want to incorporate a newly developed, desktop-based VR navigation task into their task battery and those who wish to reproduce and extend this current research. The spatial similarity task suite, as presented, provides an accessible, flexible, and powerful tool for exploring spatial memory across species. Aimed at both the research and clinical communities, this open-source toolkit, available via our GitHub repository, invites users to engage with, modify, and expand upon our foundational work. The current implementation runs on standard desktop hardware and requires minimal setup; extension to head-mounted, fully immersive VR platforms is feasible with relatively little additional effort, should researchers require greater spatial presence. This tool's significance lies in its current capabilities and in its potential for future applications with special populations. By enabling detailed examination of spatial memory, this tool can facilitate investigations into how different hippocampal subregions contribute to memory processing at various stages of development over a lifespan (Kirwan et al., 2007; Stark et al., 2013). In the visual domain, Stark and Colleagues (Stark et al., 2019, 2023) offer a highly successful analogue, the mnemonic similarity task (MST), which shares the core logic of parametric interference manipulation in a nonspatial, object-based format. Although the MST does not impose a fixed retention interval, recognition trials typically follow encoding after several intervening items, introducing a functional delay that taxes long-term memory rather than working memory processes (Yassa & Stark, 2011). The SST is particularly relevant to brain changes evident in older adults and variability in children, given the reliance of contextual memory on spatial features (Foster et al., 2012, West et al., 2023, Robin et al., 2018, Gómez & Edgin, 2016).

Age-related cognitive decline is one of the most prevalent medical issues facing our global society, with life expectancy and dementia on the rise (Nichols et al., 2022). Our VR task holds significant potential in clinical cognitive neuroscience, particularly concerning aging and the early diagnosis of memory disorders (Wolbers et al., 2014; van der Ham et al., 2015; Reggente et al., 2018; Rekers & Finke, 2024; Stramba-Badiale et al., 2024). The ability to parametrically manipulate the task demands makes our task suite well suited for assessing subtle changes in cognitive function that may precede more apparent symptoms of cognitive decline, such as those seen in mild cognitive impairment (MCI) and early Alzheimer's disease. By assessing mnemonic discrimination abilities, which are often compromised early in the development of these conditions, our task can contribute to the early detection and intervention strategies for age-related memory decline (Wiener et al., 2020; Merriman et al., 2022). A critical next step, however, is establishing baseline performance in healthy populations across the lifespan, which will provide the normative framework necessary for determining the sensitivity and specificity for future use in clinical populations. This will be essential before the task can be meaningfully adopted in diagnostic or therapeutic contexts. Ultimately, clinical validation of the SST may not only illuminate the progression of memory disorders but could open avenues for targeted interventions aimed at slowing cognitive decline in at-risk populations.

Use cases for this tool span a wide range of research areas. For example, it can be employed to study spatial skills development in children, providing insights into how these abilities evolve with age and experience. Additionally, it offers a valuable resource for exploring childhood memory disorders, such as those resulting from developmental issues or trauma where neurons are impacted in newborns (Aniol et al., 2022). This is particularly relevant considering research by Newcombe et al. (2019), that highlights the integral role of spatial skills as a foundation for cognitive skills required for education, including reasoning and mathematics. Furthermore, Newcombe (2010) emphasizes that improving spatial thinking can significantly enhance learning in math and science, underscoring the broader cognitive implications of spatial memory across development.

The task suite is also well positioned to examine how environmental factors shape memory. Researchers can design experiments to assess how different spatial environments influence memory formation and retrieval, questions that are difficult to pursue with static, screen-based assessments but tractable in a virtual environment where the space itself is a manipulable variable. The task suite is designed to be straightforward, offering researchers a customizable, open-source option for spatial memory experiments alongside existing tools. Critically, the open nature of the platform means that researchers are not limited to the tasks we have implemented. The SPA task, for instance, demonstrates how the same underlying infrastructure can support tasks with different cognitive demands (spatial recall vs. recognition). Other researchers may wish to develop tasks targeting temporal pattern separation, object-in-context memory, or navigational route learning, all of which could be implemented within this framework. Methodological consistency across laboratories and species is a prerequisite for meaningful translational progress; an open, well-documented platform is one concrete way to work toward it. Users can customize the spatial separations and size of the virtual environment, and set up a variety of experimental protocols with ease. For educators and clinicians, the suite provides a pre-prepared environmental setup and experimental protocol, ready for immediate application in classroom or clinical settings. This ease of use makes our task suite an excellent choice for conducting validated procedures without the need for extensive configuration or adaptation.

Despite its comprehensive features, we acknowledge that our toolkit has room for improvement and expansion. The inclusion of head-mounted display (HMD) technologies in future versions is anticipated to enhance the immersive experience and ecological validity of the tasks (Taube et al., 2013; Park et al., 2018; Diersch & Wolbers, 2019; Pooja et al., 2024; Kothgassner & Felnhofer, 2020). This advancement could potentially lead to more precise measurements of cognitive function by engaging users in a more realistic and engaging manner. Further developments could also include adaptive algorithms that dynamically adjust task difficulties and complexities based on real-time user performance, providing a tailored assessment environment that can better mimic real-world cognitive challenges. This collaborative approach not only facilitates ongoing refinement but also ensures that the tool evolves in line with the needs and innovations of the cognitive science community.

Our findings underscore the validity and utility of the Spatial Similarity Task suite, which was conceptually designed to align with other validated measures of pattern separation, such as the MST (Stark et al, 2019; Stark et al, 2023). While the current study did not directly assess correlations between SST performance and other behavioral tasks, a forthcoming manuscript (see Borzello, 2025, dissertation) examines the empirical relationship between the SST, the MST, and the SPA task. Preliminary analyses support several key predictions. Performance on the SST and SPA tasks is positively correlated, consistent with their shared spatial memory demands despite differing in recognition versus recall format. Performance on the SST and MST also shows positive associations, suggesting that spatial and visual mnemonic discrimination rely on overlapping interference-resolution processes. As expected, neither task correlates with the visual perception control task, which supports its validity as a non-mnemonic baseline. These findings provide important converging evidence for the utility of the SST as a tool for assessing spatial memory and mnemonic discrimination across multiple paradigms. While the resolution of interference between similar spatial locations is widely believed to depend on hippocampal computations, particularly those localized to the dentate gyrus, we acknowledge that performance on the SST could also draw on alternative memory systems, such as working memory or executive control. In particular, because the task involves a brief, uninterrupted delay between the sample and test phases (7 s), participants may employ rehearsal or strategic encoding that could dampen the relative contribution of the hippocampus. We previously piloted a version of the SST with a concurrent distractor task during the delay to mitigate such strategies; however, we removed it in the final design because the additional interference load complicated interpretation of the spatial interference manipulation and represented a departure from standard rodent spatial memory protocols.

The task suite is fully customizable and capable of incorporating longer delays or secondary tasks for researchers wishing to engage hippocampal function more directly. Increasing the delay duration followed by recall (e.g., SPA task) would likely increase reliance on hippocampal binding mechanisms and reduce reliance on working memory. The SST in its current form is optimized to assess spatial interference resolution and memory precision across the adult age span; we view it, however, as a flexible framework whose parameters can be adjusted to probe specific subcomponents of memory, including manipulations designed to more selectively recruit hippocampal processing.

In summary, the SST addresses a key challenge in cross-species translational research through the development of an open-source, desktop-based navigation paradigm for assessing mnemonic discrimination, with potential applications in early diagnosis and understanding of memory disorders (van der Ham et al., 2015; Reggente et al., 2018; Mobbs et al., 2021). Through parametric validation of the spatial distance function, which revealed a reliable memory function across close, medium, and far arm separations, and pilot data demonstrating sensitivity to interference at proximal locations, this task delivers reliable and reproducible findings that will enrich our understanding of spatial memory across both healthy and clinical populations. Critically, the task's validation against the established MST, demonstrating a significant positive correlation between SST and MST performance alongside expected dissociations from the visual perception control task, provides convergent support for the SST as a measure of spatial mnemonic discrimination. As the field moves toward standardized, open-source cognitive tools that can be deployed across laboratories, species, and clinical contexts, the SST suite represents a concrete step in that direction.

## Supplementary Information

Below is the link to the electronic supplementary material.

Supplementary file1 (DOCX 347 KB)

Supplementary file2 (DOCX 1363 KB)

## Data Availability

Anonymized sample dataset and materials are available in a GitHub repository: https://github.com/Spatial-Similarity-Task/SST.

## Code availability

Experiment code and analysis code for the example study are available in a GitHub repository: https://github.com/Spatial-Similarity-Task/SST.
