# Supplementary material for: The Spatial Similarity Task: A cross-species approach to spatial memory
Source: Behav Res Methods. 2026 Aug 18;58(9):273. doi: 10.3758/s13428-026-03127-5 (PMC13486031; doi:10.3758/s13428-026-03127-5)
Supplement: Supplementary file 1 [file 13428_2026_3127_MOESM1_ESM.docx]

**Spatial Similarity Task (SST) Protocol**

MATERIALS

*Equipment/software*

- xbox controller (e.g. Xbox Wireless Controller + USB-C® Cable)
- Unity suite installed on Windows PC
- Monitor

[1] Experimental set up and procedure

1) Preparation before running a subject
   a) Create three separate arm combination schedules for training, practice, and task modules. Save as CSV files and put files in Schedules folder. Schedule filenames should be as follows:
      i) SPS_FullTask_Schedule.csv
      ii) SPS_Practice_Schedule.csv
      iii) SPS_Verbal_Training_Schedule.csv
      iv) SPS_NonVerbal_Training_Schedule.csv
   b) For example, if the Unity build file is kept here (path: C:\Users\lab\Spatial Similarity Task\SST Build), then the schedule file being used should be saved in the Schedule folder here: C:\Users\lab\AppData\LocalLow\CognitiveScience\Spatial Similarity Task\Trial Logging\Schedule
2) Before each experimental session, turn on the computer and the testing monitor.
   a) Connection of Xbox controller and testing- Ensure that the Xbox controller is connected to the computer and works. *Use* *AntiMicro*for this.
   b) Make sure you have printed the consent forms ready.
   c) Have questionnaires in printed form or on Qualtrics ready.
      i) Info sheet
      ii) Pre-Task Questionnaire
      iii) Post-Task Questionnaire
      iv) Navigational strategies questionnaire
      v) SBSOD questionnaire
3) Intro and consent procedure
4) Ask the participant to sit in the experimental chair and explain the experimental procedure according to the prepared “Experimenter script” in the following pages (you can say the script word for word or in your own words).
5) Administer Pre-task questionnaire.
6) Inform the participants that they can ask the experimenter questions regarding the questionnaire at any time and can skip any questions they don’t feel comfortable answering.
7) Prepare Spatial Similarity Task suite
   a) When ready to run task with subject, make sure subject is the correct distance from the monitor- use the string (~49.5 inches) attached to the monitor base and stretch string to the edge of the chair back. The end of the string should be where the chair back is.
   b) On the experimenter monitor, open the executable Unity file for the experiment. For example, let’s say the Unity build file is kept here (path: C:\Users\lab\SST\SST Build), then within the SST Build folder, double click the Spatial Similarity Task Unity icon.

[2] To run the Spatial Similarity Task suite

1) Test Settings

This is the first GUI that will pop up. Here you’re able to set the timings for the task.

The default timings are shown below:

| ITI (black screen) | .25 seconds |
| --- | --- |
| Fixation | .5 seconds |
| In start box | 2 seconds |
| Sample phase | 45 seconds max* |
| Blank screen/delay | .25 seconds |
| ISI | 7 seconds |
| Test phase | 45 seconds max* |
| Total | 100 seconds max |

2) Main menu- Welcome to the Spatial Similarity Task. We are about to begin.

This is where you create a filename and set the path to the output data logs for each level. After each level, you’ll return back to this main menu page.

| Main Menu Field |  |
| --- | --- |
| Levels | - Baseline (Baseline Task) - Training (Verbal Training - SPS) - Training 2 (non-verbal) - Practice (Verbal Practice - SPS) - SPS Task (Full Task – SPS) (version w/o arms) - SPS Task 2 (version w/ arms or clear walls) |
| Logfile directory | This is the path to where the data gets saved |
| Subject ID | Input the unique ID code of the subject |
| Session | Default is 1. Can input session number if subject runs the task more than once and you want to save both experimental outputs separately |
| Date | Type of the today’s date/recording date  Convention: YEARMMDD , with the year (YEAR) first, then month (MM), and then the day (DD). |
| Switch scale | Allows user to use a virtual maze at rat-sized scale (“rescaled”) or a larger size maze (“Original scale”) |

You should through the levels in this order: _Baseline_ → _Training_	→ _Practice block_	→ _SPS task_

| Module | Comments |
| --- | --- |
| All | - After you fill in the fields and are ready to begin the experiment, click "Begin Session". When you press “Begin Session”, you must press the spacebar to start for each module of the task. - To prevent automatic overwriting, a popup will appear if filename you create already exists in data folder, asking if you want to overwrite. - XBOX controller assignments:      - Keyboard shortcuts:   U - force quit/exit/return to main menu  P - pause  O - skip a trial  I - skip phase of a trial |
| Baseline | - Before exiting the Baseline, you can show subjects an extra functionality- the ability to run. To increase speed with controller, hold down the left stick button |
| Practice | - If subject scores 50% or lower in practice block, the experimenter can opt to repeat the training and/or practice modules. - If subject scores above 50% in practice block, the subject is likely ready to begin the task, but it's suggested that the experimenter check in with subject before starting task to see if there are any questions/clarifications. |

[3] Administer the three Post-task questionnaires

1) Post-task questionnaire,
2) Navigational Strategies questionnaire (NSQ),
3) And Santa Barbara Sense of Direction Scale questionnaire (SBSOD)

[4] After each experimental session

1) Inform the participant that the experimental part is now finished. Thank her or him for participating in the experiment. Ask if the participant has any questions regarding the purpose of the experiment, and if not, then walk him or her out of the lab.
2) Check that output logs were saved in the logfile directory set in the main menu
   a. Outputs = files, folders, and screen recordings
   b. For example, if the Unity build file is kept here: C:\Users\lab\Spatial Similarity Task\SST Build
   c. The output data log files and folders are saved here: C:\Users\lab\AppData\LocalLow\CognitiveScience\Spatial Similarity Task\
