# Supplementary material for: The Spatial Similarity Task: A cross-species approach to spatial memory
Source: Behav Res Methods. 2026 Aug 18;58(9):273. doi: 10.3758/s13428-026-03127-5 (PMC13486031; doi:10.3758/s13428-026-03127-5)
Supplement: Supplementary file 2 [file 13428_2026_3127_MOESM2_ESM.docx]

**Supplementary Information**

A. Full Protocol- [Protocol.docx](https://ucsdcloud-my.sharepoint.com/:w:/r/personal/mborzell_ucsd_edu/Documents/paper%20drafts/methods%20paper/Protocol.docx?d=wa23da72c957f4316aff6bd1c29283e0d&csf=1&web=1&e=pxyyKY)
B. Questionnaires

Pre-task questionnaire

Post-task questionnaire

Navigational Strategies Questionnaire (NSQ)

Santa Barbara Sense of Direction Questionnaire (SBSOD)

C. Supplemental Tables

Supplemental Table 1. Configurable parameters of the Spatial Similarity Task suite

| **Parameter** | **Options** | **How to Adjust** |
| --- | --- | --- |
| Number of trials | Any number | Unity GUI |
| Arm separations | Any subset of 25 possible separations | Unity GUI |
| Trial timing (sample phase, delay, test phase) | Fully adjustable | Unity GUI (see Supplemental Figure 5) |
| Maze design | With arms / open field (arms removed) | Unity GUI |
| Maze size | Rat-scaled / large environment | Unity GUI |
| Display type | Standard flat monitor / ultrawide curved monitor | Hardware selection |
| Training type | Verbal instructions / non-verbal (observational) | Unity GUI |

Supplemental Table 2. Troubleshooting Table

| **Problem** | **Solution** |
| --- | --- |
| Poor performance in practice module | - To prevent misunderstandings, check in with participant throughout- ask if they understand the instructions. Pay attention to the practice session as the scores will tell you if the participant understood the task and what he should be doing (e.g., 4 out of 5 is considered good). - Ask the participant if she would like to re-do the practice (e.g., “The task can be a bit tricky, but you will get the hang of it! It’s fast, so it’s understandable if you miss some trials. Let's try doing that practice session again and then continue with the rest of the task.”) |
| Weird viewpoint | - When you start a particular level, sometimes the viewpoint is pointed towards the ground and looks like a blank screen so you may need to adjust the controller to see everything. - Make sure the start screen is not facing the ground. |
| New trial is not starting | - If this happens during the Training module, you may need to mention to participants that when returning to the start box, they need to go all the way into the start box for a new trial/phase to begin. |
| Start box door is not opening | - This means that there is no schedule csv file in the Schedule folder. |
| Overwrite function | - To prevent automatic overwriting, a popup will appear if the filename you want to create already exists in the data folder, asking if you want to overwrite. - For the “Session,” put “2”. Otherwise, you will overwrite the “Full Task” data from the other maze. - If you decide to overwrite, both sessions will be saved in the file- the first part of the file corresponds to the session ran initially followed by the data from the second session. |
| Software issue | - In case of having to rerun the task because computer crashes or Unity is bugging-quite the task and restart again but change the “Session” number (e.g., put 2) so you do not accidentally overwrite anything. - Check physical connects, reboot the system |

D. Supplementary Figures

**Supplemental Figure 1. Photograph of our lab set up at UCSD (University of California San Diego) showing two testing stations.** Each testing station has an ultrawide, bent monitor for the participant and an experiment monitor.

**Supplemental Figure 2. Schematic of Arm Separations and Combinations.** Illustration of the different arm separation distances and pairings available to the experimenter when configuring spatial similarity trials.

**Supplemental Figure 3. Data logging file and folder structure.** Overview of the file organization used to save task performance and behavioral data.

**Supplemental Figure 4. Screenshot of data logging files that are created.** (A) CSV file with overall measures that can be obtained and (B) CSV file from a single trial.

**Supplemental Figure 5. Task Settings Interface for Custom Timing Input.** Graphical user interface (GUI) where the researcher inputs custom timing parameters for each phase of the task, allowing flexible control over trial structure.

**Supplemental Figure 6. Spatial Association Task.** Similar to the SST, each trial of the SPA task consists of a sample phase, a brief delay period, and a test phase.
